# Nomogram Based on Dual-Layer Spectral Detector CTA Parameter for the Prediction of Infarct Core in Patients with Acute Ischemic Stroke

**DOI:** 10.3390/diagnostics13223434

**Published:** 2023-11-13

**Authors:** Yan Gu, Dai Shi, Hao Shen, Yeqing Wang, Dandan Xu, Aoqi Xiao, Dan Jin, Kuan Lu, Wu Cai, Liang Xu

**Affiliations:** 1 Department of Radiology, The Second Affiliated Hospital of Soochow University, Suzhou 215000, China; gy251997@163.com (Y.G.); shidai318@163.com (D.S.); tymyrcl@163.com (Y.W.); xdd4860@163.com (D.X.); xaq7687@163.com (A.X.); jindan0904@163.com (D.J.); lukuan1011@163.com (K.L.); xwg608@126.com (W.C.); 2 Department of Medical Oncology, Anhui Provincial Hospital Affiliated to Anhui Medical University, Hefei 230000, China; shenhaoahmu@163.com

**Keywords:** acute ischemic stroke, dual-layer spectral detector CT, iodine density values, infarct core, prediction model

## Abstract

(1) Background: Acute ischemic stroke (AIS) is time-sensitive. The accurate identification of the infarct core and penumbra areas in AIS patients is an important basis for formulating treatment plans, and is the key to dual-layer spectral detector computed tomography angiography (DLCTA), a safer and more accurate diagnostic method for AIS that will replace computed tomography perfusion (CTP) in the future. Thus, this study aimed to investigate the value of DLCTA in differentiating infarct core from penumbra in patients with AIS to establish a nomogram combined with spectral computed tomography (CT) parameters for predicting the infarct core and performing multi-angle evaluation. (2) Methods: Data for 102 patients with AIS were retrospectively collected. All patients underwent DLCTA and CTP. The patients were divided into the non-infarct core group and the infarct core group, using CTP as the reference. Multivariate logistic regression analysis was used to screen predictors related to the infarct core and establish a nomogram model. The receiver operating characteristic (ROC) curve, the calibration curve, and decision curve analysis (DCA) were used to evaluate the predictive efficacy, accuracy, and clinical practicability of the model, respectively. (3) Results: Multivariate logistic analysis identified three independent predictors: iodine density (OR: 0.022, 95% CI: 0.003–0.170, *p* < 0.001), hypertension (OR: 7.179, 95% CI: 1.766–29.186, *p* = 0.006), and triglycerides (OR: 0.255, 95% CI: 0.109–0.594, *p* = 0.002). The AUC–ROC of the nomogram was 0.913. Calibration was good. Decision curve analysis was clinically useful. (4) Conclusions: The spectral CT parameters, specifically iodine density values, effectively differentiate between the infarct core and penumbra areas in patients with AIS. The nomogram, based on iodine density values, showed strong predictive power, discrimination, and clinical utility to accurately predict infarct core in AIS patients.

## 1. Introduction

Stroke is the leading cause of disability and death in adults worldwide, and the prevalence and incidence of strokes in China are on the rise [1]. In 2019, there were 3.94 million new stroke cases in China [2]. In the last decade, the incidence of stroke in China has increased by 86%, with about 3.94 million new cases, ranking first in the world [2]. Acute ischemic stroke (AIS) is the main type of stroke, and is characterized by dangerous onset and high morbidity, recurrence, and mortality rates, which has imposed a heavy economic burden on society and families. Statistics showed that the direct medical cost of AIS in our country reached CNY 50 billion in 2015 [3]. In clinical practice, bridging intravenous thrombolysis (IVT) may be more beneficial than direct endovascular thrombectomy (EVT) in patients with rapid core growth [4]. Thus, it is essential to use appropriate examination methods to promptly identify the infarct core.

In the present day, AIS is mainly diagnosed through imaging studies, such as head computed tomography (CT) and computed tomography angiography (CTA) [5]. CTA is an examination that combines CT enhancement technology with thin-layer, wide-area, and fast scanning technology to diagnose vascular diseases by injecting contrast agents through a peripheral vein, acquiring data from CT scanning, and imaging them using a special post-processing method, which provides an accurate and effective all-around view of intracranial blood vessels. Computed tomography perfusion (CTP) is functional imaging that evaluates the perfusion status of brain tissues by performing continuous dynamic scanning of selected levels of interest, obtaining temporal density profiles of each pixel of the selected levels and processing them through mathematical modeling to obtain hemodynamic parameters and perfusion images. Although brain CTP benefits from the use of automated software, challenges associated with it remain, such as technician training, delay in acquisition time, measurement error in the infarct core and penumbra, motion sensitivity, and the risk of additional radiation doses. It has been shown that 7% of perfusion images in AIS patients cannot be analyzed by post-processing software. Diffusion-weighted imaging (DWI) and positron emission tomography (PET) are too time-consuming and difficult to obtain, so they are not the most applicable method for detecting AIS in current medical conditions. Therefore, a new imaging diagnostic technique needs to be explored. Dual-layer spectral detector computed tomography (DLCT) is a scanning scheme based on the separation of high- and low-energy photons via a dual-layer detector proposed in recent years, which utilizes different crystalline scintillating substances in the upper and lower layers of a three-dimensional dual-layer detector to directly absorb different energy rays, realizing the “homologous, simultaneous, and homonymous” energy spectroscopy technique, which advances energy spectroscopic CT from the exploratory stage to mature clinical application [6].

Currently, spectral CT has developed rapidly, is unique, and has more advantages in head and neck angiography. Spectral parameters, such as iodine density value and effective atomic number (Zeff) value, have great application potential in stroke diagnosis. At present, there have been no foreign or domestic reports of a nomogram model for predicting the infarct core in AIS. Therefore, this study aimed to investigate the application potential of DLCTA in differentiating infarct core from penumbra in patients with AIS and to establish a simple and reliable nomogram combining spectral CTA parameters to predict the probability of infarct core in AIS patients.

## 2. Materials and Methods

### 2.1. Patient Selection

The imaging and clinical data of 102 patients with AIS, diagnosed between March 2022 and December 2022 in our hospital, were collected and analyzed retrospectively. The inclusion criteria were as follows: (1) diagnosis with acute ischemic stroke and examination via dual-layer spectral detector CTA and CTP; (2) onset time < 12 h; (3) clear images without artifacts and complete clinical data; and (4) male or female, aged 18–90 years. The exclusion criteria were as follows: (1) comorbidities, such as a brain tumor, blood system disease, severe organ failure, or other life-threatening disease and (2) previous history of major cranial trauma or intracranial surgery. The study was approved by the Ethics Committee of our hospital (JD-HG-2022-69).

### 2.2. Imaging Acquisition

**Figure 1:**
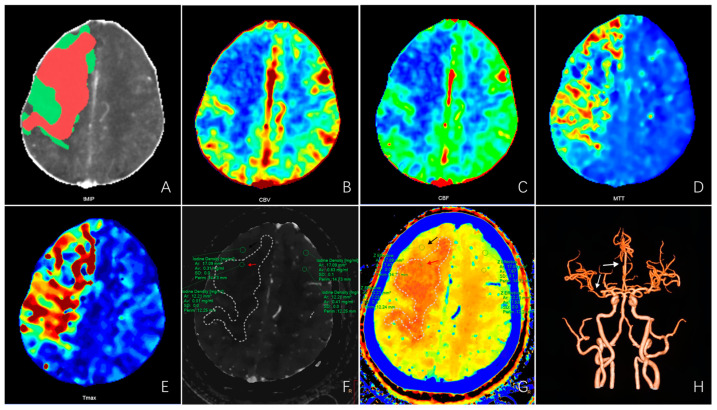
CTP and spectral CTA images of a patient with acute ischemic stroke. A 75-year-old female with a history of hypertension was admitted with sudden onset of left limb weakness with aphasia for 3 h (NIHSS 13). (**A**) CTP post-processing image shows the infarct core (red area) and penumbra (green area). (**B**–**E**) The CBV, CBF, MTT, and Tmax maps of CTP parameter images show perfusion defects in the right frontoparietal occipital lobe. CBV and CBF were lower than the contralateral side; MTT and Tmax were longer than the contralateral side. (**F**,**G**) The iodine density map and the effective atomic number map of spectral CTA show perfusion defects in the right frontoparietal and occipital lobe. The iodine density values in the infarct core area (red arrow) and penumbra area (black arrow) were 0.01 mg/mL and 0.31 mg/mL, respectively, which were significantly lower than the contralateral side (0.41 mg/mL and 0.63 mg/mL). The effective atomic number values in the infarct core and penumbra were 7.23 and 7.47, respectively, which is significantly lower than the contralateral region (7.53 and 7.67). (**H**) CTA image shows the right M1-MCA and A2-ACA occlusion (white arrow). Abbreviations: CBV—cerebral blood volume; CBF—cerebral blood flow; MTT—mean transit time; Tmax—time to top; M1-MCA—the M1 segment of the middle cerebral artery; A2-ACA—the A2 segment of the anterior cerebral artery.

An IQon spectral CT machine (Philips, the Netherlands) was used to perform head and neck CTA and brain CTP imaging (Figure 1).

Patients laid supine, and the head was immobilized with a fixation plate before scanning, keeping the head still and avoiding swallowing movements.

CTP: Axial scanning mode was used. The scanning range was 8 cm above the cavernous sinus. Iopamidol (370 mg/mL (dose 40 mL)) and saline (18 mL) were administered intravenously via the right elbow (5 mL/s). Exposure was started 3 s after contrast medium injection and performed 15 times in 60 s without interval.

CTA: Spectral spiral scanning mode was adopted. The ROI was placed at the level of the descending aorta, and the scan ranged from the aortic arch to the roof of the skull. CTP scanning procedure was followed by iopamidol (370 mg/mL (dose 50 mL)) and saline 50 mL. When the contrast medium concentration in the target vessel peaked after threshold triggering(120 Hu), it automatically switched to spectral CTA scanning mode. Table S1 shows the specific scanning parameters.

### 2.3. Data Collection

Post-processed images from CTP examinations were obtained using brain perfusion analysis software from Philips Healthcare. In these images, the red area indicates the infarct core, representing “irreversibly” affected brain tissue with rCBF < 30% [7], and the green area indicates the penumbra, representing “essentially reversibly” affected ischemic tissue and corresponding to the area around the infarct nucleus where nerve cells were ischemic and dysfunctional but energy metabolism was preserved [8]. ROIs in the cerebral perfusion defect areas (infarct core, penumbra) were delineated manually first, and ROIs in the contralateral mirror brain area were then automatically obtained. CTA iodine density and Zeff maps were obtained at the Philips SpDS image and PACS workstations via spectral base image packets. Referring to the perfusion defect areas (infarct core, penumbra) obtained via CTP, we identified and delineated the ROIs of the affected and unaffected sides at the corresponding levels and sites of conventional thin-sliced spectral CT images. Then, we switched to the iodine density and Zeff maps to measure and record the iodine density and Zeff values of the affected and unaffected sides.

The following clinical data of patients were collected by consulting the electronic record system: admission Glasgow Coma Scale (GCS) score; drinking history; smoking history; sex; age; admission-modified Rankin Scale (mRS) score; admission National Institutes of Health Stroke Scale (NIHSS) score; lesion location (anterior circulation, posterior circulation); Trial of Org 10172 in Acute Stroke Treatment (TOAST) classification (large-artery atherosclerosis (LAA), cardioembolism (CE), etc.); risk factors (previous history of stroke, hypertension, diabetes, cancer, paralysis, chronic heart failure, coronary heart disease, atrial fibrillation, pulmonary infection) and other basic data; leukocyte, platelet, lymphocyte, neutrophil, and monocyte counts; and C-reactive protein (CRP), high-density lipoprotein (HDL), low-density lipoprotein (LDL), cholesterol, triglyceride, and glycosylated hemoglobin (HbA1c) levels, among other laboratory parameters.

### 2.4. Statistical Analysis

Data analysis was performed using SPSS V.26.0, GraphPad Prism V.9.0, and R software V. 3.6.1. Continuous variables are presented as the mean with standard deviation (x ± s) or median with interquartile range (M (Q1, Q3)). Comparisons between groups were performed using an independent samples *t*-test or Mann-Whitney U test. Categorical variables are reported as numbers and percentages (n%) and were analyzed using the χ^2^ test. The cut-off value for iodine density and atomic number was determined using GraphPad Prism v.9.0 software. Specifically, a receiver operating characteristic (ROC) curve was generated using the iodine density values of all samples, and the optimal cut-off value was determined based on the Youden index, which maximizes the sum of the true positive rate and true negative rate. Univariate and multivariate logistic regression analyses were performed to identify independent risk factors associated with the infarct core in AIS; then, the rms package was used to establish a nomogram model of the infarct core in patients with AIS. The area under the receiver operating characteristic (ROC) curve (AUC) was used to assess the predictive accuracy of the nomogram model. Calibration curves and decision curve analysis (DCA) were used to evaluate the calibration and clinical effectiveness of the model, respectively.

## 3. Results

### 3.1. Patient Characteristics

The present study included 102 AIS patients, with an average age of 64.4 ± 13.07 years (range, 28–89 years). Patients were divided into the infarct core group (n = 67) and the non-infarct core group (n = 35), using CTP as the reference. A comparison of the two groups showed significant differences in the iodine density value, Zeff value, neutrophil count, and triglyceride level (all *p* < 0.05) (Table 1 and Table 2).

### 3.2. Cut-Off Values for Iodine Density and Effective Atomic Number

**Figure 2:**
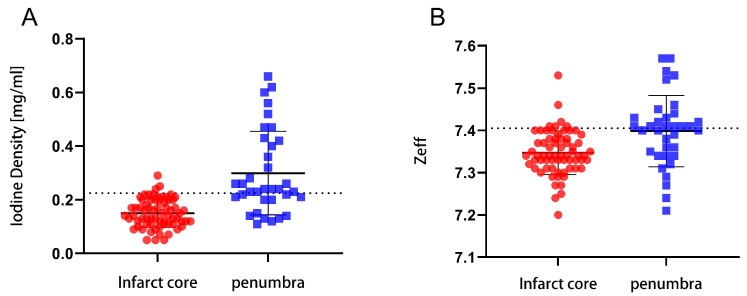
The optimal cut-off points for the iodine density and effective atomic number in the infarct core and penumbra areas. (**A**) The optimal cut-off points for the iodine density values. (**B**) The optimal cut-off points for the effective atomic number values.

The cut-off values for the iodine density and effective atomic number in the infarct core and penumbra areas were 0.225 mg/mL and 7.405, respectively (Figure 2). This indicates that the iodine density and effective atomic number values have the ability to distinguish the infarct core from the penumbra.

### 3.3. Univariate and Multivariate Logistic Analyses of Clinical and Spectral CT Parameters

Significant independent risk factors identified in the univariate logistic regression analysis (*p*-values were relaxed to 0.2 to avoid missing some important factors) were included in the multivariate logistic regression analysis. The results showed that the iodine density, presence of hypertension, and triglyceride level were independent predictors of the infarct core in AIS (OR (95% CI) of 0.022 (0.003–0.170), 7.179 (1.766–29.186), and 0.255 (0.109–0.594), respectively) (all *p* < 0.05) (Table 3).

### 3.4. Development and Performance of the Prognostic Nomogram

**Figure 3:**
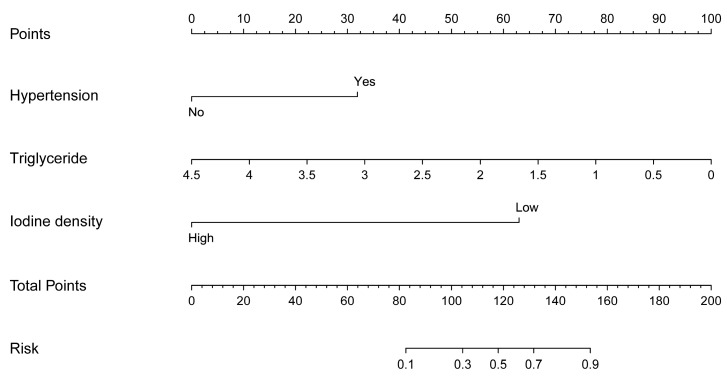
Nomogram predicting infarct core in patients with acute ischemic stroke.

**Figure 4:**
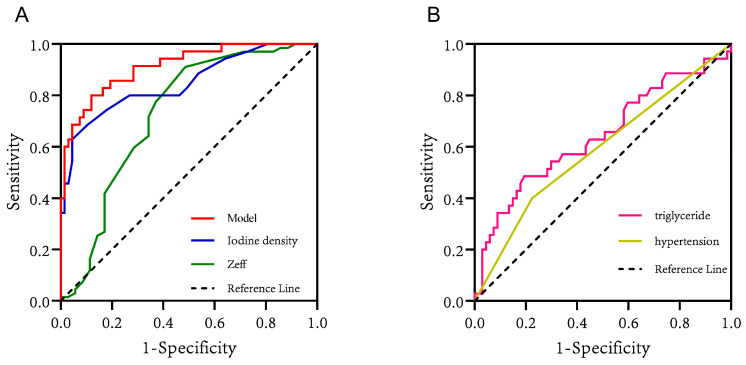
(**A**) The ROC curves of iodine density values, effective atomic number values, and the model for predicting infarct core in patients with AIS. (**B**) The ROC curves of triglycerides and hypertension for predicting infarct core in patients with AIS.

**Figure 5:**
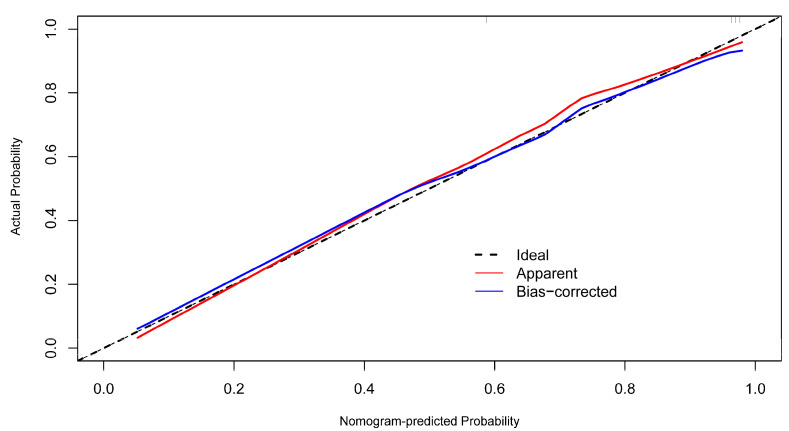
The calibration curves for the nomogram.

**Figure 6:**
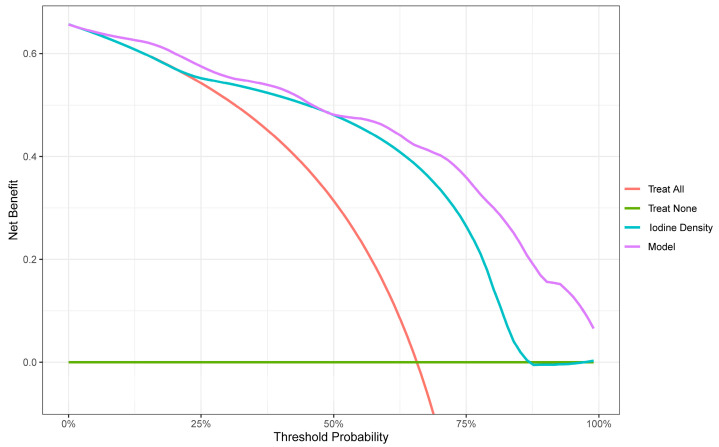
Decision curve analysis (DCA). The net benefit is shown on the y-axis, and the threshold probability is shown on the x-axis. Use of the model (violet line) achieves the highest net benefit compared with the iodine density values (blue line), treat-all strategy (red line), and treat-none strategy (horizontal green line).

The nomogram prediction model for the infarct core in AIS consisted of three independent risk factors (Figure 3). Positioning on the horizontal axis corresponds to the range of each factor, and positioning on the fractional vertical axis corresponds to the score of each factor. The sum total score of each factor corresponds to the point on the infarct core risk axis, which is the probability of the patients having an infarct core. The area under the ROC curve (AUC) of this model was 0.913 (95% CI: 0.855–0.971), with a sensitivity of 80% and a specificity of 88.06%, indicating the good discrimination and predictive accuracy of the model (Figure 4). The calibration curve of this model coincided well with the actual curve, showing good agreement between the predicted probability of an infarct core in AIS by the model and the actual probability, with a high degree of calibration (Figure 5). DCA was used to evaluate the clinical validity of the model, and the results indicated that clinical intervention according to the nomogram could provide better clinical benefits in the vast majority of patients when the threshold probability was in the range of 0 to 1.0. This predictive model showed a high net benefit at almost all threshold probabilities, indicating good clinical utility (Figure 6).

## 4. Discussion

Stroke is the second leading cause of death worldwide, after ischemic heart disease, and is the leading cause of disability and death among adults in China [9,10]. Acute ischemic stroke accounts for 87% of all strokes and is characterized by high morbidity, recurrence, and mortality rates [11]. Critical to AIS treatment is salvaging the reversible penumbra and delaying infarct core progression. Intravenous thrombolysis provides benefits within 4.5 h post-onset, requiring imaging for core and penumbra assessment when onset exceeds 4.5 h. Overestimating infarct volume may exclude patients from reperfusion, and underestimating it may increase bleeding risk, resulting in adverse outcomes [12]. Accurate core and penumbra assessment guides treatment decisions for precision and individualized care, ensuring reliability, safety, and cost-effectiveness in clinical diagnosis and treatment. The accurate identification of the irreversible infarct core and rescuable ischemic penumbra areas in AIS patients is an important basis for formulating treatment plans, so timely diagnosis is essential to guide clinical decision making.

At present, the early identification and diagnosis of AIS is mainly made by adopting imaging techniques. PET imaging is considered the gold standard for AIS diagnosis [13], and DWI is highly recognized in assessing the accuracy of AIS size [14]. However, PET and DWI imaging are time-consuming and inconvenient, and PET examination procedures are complex, invasive, and radioactive, making them unsuitable for application in routine clinical settings for AIS patients [15,16]. CTP is currently the most important diagnostic method for the assessment of infarct core and penumbra in patients with AIS, but its applicability and accuracy in AIS assessment have been questioned due to its large radiation dose, long scanning time, examination failure due to poor immobilization in some patients, and the frequent overestimation and underestimation of infarct volume [17,18,19]. Due to the lack of standardized post-processing protocols, the measurement of infarct core volume varies widely among CTP algorithms [20]. In addition, an incorrect CTP protocol may increase the radiation dose to organs, including skin and lens, by 3- to 11-fold [21]. Therefore, we need to find a new diagnostic technique that is safer and more reliable.

In recent years, the introduction of spectral CTA has provided new imaging techniques for AIS. Compared with brain CTP, dual-detector spectral CTA has the advantages of optimized process, low radiation dose, low contrast medium dose, high image quality, accurate energy analysis, and no scan field restriction. It employs simultaneous energy separation scanning, reducing motion artifacts. No special program of pre-scanning setting is required, and spectral parameters are stored for on-demand analysis [6,22]. It also provides iodine density and Zeff maps in a color-quantified format to improve the visualization of lesion tissues and enrich the diagnostic means. This approach combines anatomical and functional data in a single scan, offering insights into vessel structure, plaque composition, and blood perfusion. Therefore, DLCTA can enable a “one-stop” assessment of the severity of brain tissue perfusion defects, intracranial vascular stenosis, or occlusion and is expected to provide a safer and faster method for assessing AIS patients.

Spectral CTA concurrently acquires multiparameter images, with the iodine density map highlighting iodine distribution, leveraging the spectral contrast between iodine and water. This aids in quantifying iodine enhancement, enhancing visualization in contrast-enhanced tissues, and improving perfusion defect detection sensitivity in AIS. Iodine density values directly assess brain tissue perfusion, making it a sensitive indicator. This approach outperforms traditional CTP, which is less sensitive to smaller subcortical infarcts [23,24,25]. Previous studies have found that iodine quantification measured via spectral CT is reliable even at low concentrations of iodinated contrast agents [26,27,28]. Additionally, combining iodine density maps with energy spectrum curves and effective atomic number maps can identify carotid atherosclerotic plaque composition and assess its relationship with stroke occurrence for tailored treatment strategies.

Some scholars have studied the application of spectral CT in evaluating blood perfusion in myocardial, liver, pancreas, kidney, and other organ lesions and confirmed that there is some correlation between iodine density values and some parameters of CTP [29,30,31,32]. Fransson et al. [33] showed that quantitative analysis of iodine density could separate normal and perfusion defect tissues. In this study, we further measured that the iodine density value was lower in the infarct core area than in the penumbra area (0.14 (0.11, 0.20) vs. 0.24 (0.20, 0.42), *p* < 0.001), with a cut-off value of 0.225 mg/mL, indicating that the iodine density value can reflect the perfusion difference between the infarct core area and penumbra areas to a certain extent. The results of this study showed that iodine density value and the presence of an infarct core in patients with AIS were statistically significant in both univariate and multivariate analyses, and that the iodine density value was an independent risk factor for infarct core, which has not been seen in previous studies. In this study, we further emphasized the diagnostic value of iodine density values in AIS, not only for the ability to distinguish the infarct core area from the penumbra area but also to predict the probability of the presence of an infarct core area in AIS patients. The iodine density value had an AUC of 0.848, a sensitivity of 62.86%, and a specificity of 95.52% for the discrimination of infarct core and penumbra area, indicating that the iodine density value has a high clinical value in identifying AIS infarct core.

The Zeff value reflects the composition of substances and indirectly reflects changes in the blood supply of tissues. Huang et al. [34] found that the spectral CT parameters iodine density value and Zeff value can effectively distinguish the perfusion defect area from normal brain tissue in AIS patients. In this study, the Zeff value had an AUC value of 0.726, a sensitivity of 51.43%, and a specificity of 91.04% for distinguishing the infarct core from the penumbra area. When the Zeff value was below 7.405 (cut-off), a high possibility of an infarct core was demonstrated. However, multivariate analysis showed that the Zeff value was not statistically significant for the presence of infarct core in AIS patients, so more studies are needed to explore the correlation.

In our study, besides the well-known spectral parameters (iodine density values) as the strongest predictors for infarct core, we found that hypertension and triglycerides at admission were also independently associated with the infarct core based on our extensive collection of clinical data. The AUC values of 0.588 (sensitivity: 40%; specificity: 77.61%) for hypertension and 0.642 (sensitivity: 48.57%; specificity: 80.60%) for triglycerides indicated that they have certain discriminatory and predictive values. Furthermore, both of them were statistically significant in univariate and multivariate analyses. As we know, there are many risk factors for stroke, among which hypertension (52%) is considered the most common modifiable risk factor [35]. Studies have shown that hypertensive patients have larger infarcts and less salvageable tissue than normotensive patients [36]. Our study came to a similar conclusion that hypertension was positively correlated with AIS infarct core progression, and brain tissues in hypertensive patients were more likely to suffer from cerebral infarction under the stimulation of ischemia. Many studies have found that hypertension aggravates infarct core progression and worsens the outcome of stroke, mainly through mechanisms such as collateral circulation damage, blood–brain barrier destruction, white matter damage, and edema formation, as well as brain self-regulation impairment [36,37,38]. Triglyceride levels have been reported to be negatively associated with stroke outcomes, with a low triglyceride level being associated with an increased infarct volume on brain CT at admission, increased stroke severity, and increased mortality after stroke [39,40]. In the present study, the triglyceride level was also found to be strongly associated with infarct core progression in AIS, and the nomogram risk prediction model showed that a lower triglyceride level resulted in a higher score and, therefore, a greater probability of having an infarct core. Systematic reviews have reported that high triglyceride levels protect against AIS mainly by providing nutrition to the brain tissue of hypermetabolic stroke patients and as a buffer of lipotoxicity via high concentrations of unsaturated fatty acids in the brain to reduce the effects of oxidative stress [41,42].

To further improve the accuracy of the spectral parameter iodine density value in properly predicting infarct cores in patients with AIS, we developed a prediction model combining the above factors: the iodine density value as a spectral imaging parameter, the presence of hypertension as a clinical indicator, and the triglyceride level as a laboratory indicator. These indicators reflect the comprehensive and multidimensional nature of the model. Furthermore, hypertension and triglyceride levels are simple and easily obtained clinical parameters; thus, the practicability of the model is high, which has obvious advantages in the early detection of infarct core in AIS. Compared with individual iodine density values, hypertension, and triglycerides, the nomogram model was better at predicting the risk of infarct core, showing a higher C index of 0.913 (95% CI: 0.855–0.971), with a sensitivity of 80% and a specificity of 88.06%, indicating that the model had a good discriminatory and predictive efficacy. The calibration curve of this model coincided well with the actual curve, showing good agreement between the predicted probability of an infarct core in AIS by the model and the actual probability, with a high degree of calibration. The DCA curve showed that this predictive model had a high net benefit at almost all threshold probabilities, indicating good clinical utility.

Our study had several limitations. First, this was a single-center study with a relatively small number of patients, and multicentre and multicenter studies with large sample sizes are needed in the future. Second, this study used a retrospective method to collect medical records, making bias inevitable in the collection process. Third, our study did not validate the credibility of this model on an independent dataset, and patient data should be collected from different centers during the same time period in the future to establish an independent dataset for validation to assess its general applicability.

## 5. Conclusions

Spectral CT parameters, specifically, iodine density values, can effectually distinguish infarct core from penumbra regions in patients with AIS. The nomogram model combined with the spectral CT parameter demonstrates an excellent predictive effect, differentiation, and clinical utility in the timely clinical prediction of the infarct core area. With further validation, the model could potentially be a reliable and intuitive visual guidance tool.

## Figures and Tables

**Table 1:** Spectral parameters of the infarct core group and the non-infarct core group.

Variables	Infarct Core Group (n = 67)	Non-Infarct Core Group (n = 35)	Test Statistic	*p*-Value
Iodine density (mg/mL)	0.14 (0.11, 0.20) ^a^	0.24 ± (0.20, 0.42) ^c^	5.406	<0.001 *
Zeff ^a^	7.34 (7.31, 7.38)	7.41 (7.34, 7.43)	2.394	0.021 *

Zeff—the effective atomic number. ^a^ represents the Z value analyzed by Mann-Whitney U test, and ^c^ is t value analyzed by Student’s *t*-test. * represents *p* < 0.05.

**Table 2:** Clinical characteristics of the infarct core group and the non-infarct core group.

Variables	Infarct Core Group (n = 67)	Non-Infarct Core Group (n = 35)	Test Statistic	*p*-Value
Gender ^b^			0.989	0.320
Male	52 (68.4)	24 (31.6)		
Female	15 (57.7)	11 (42.3)		
Age (years) ^b^			0.080	0.778
<70	44 (66.7)	22 (33.3)		
≥70	23 (63.9)	13 (36.1)		
Smoking ^b^	34 (63)	20 (37)	0.378	0.539
Drinking ^b^	25 (65.8)	13 (34.2)	0	0.987
GCS ^c^	13.09 ± 2.57	12.94 ± 2.71	0.269	0.789
Pre-stroke mRS ^b^			0.124	0.725
0–2	32 (64)	18 (36)		
3–6	35 (67.3)	17 (32.7)		
Baseline NIHSS ^b^			2.195	0.138
<15	42 (60.9)	27 (39.1)		
≥15	25 (75.8)	8 (24.2)		
Lesion location ^b^			0.835	0.361
Anterior	57 (64)	32 (36)		
Post	10 (76.9)	3 (23.1)		
TOAST classification ^b^			0.203	0.903
LAA	48 (66.7)	24 (33.3)		
CE	8 (66.7)	4 (33.3)		
Others	11 (61.1)	7 (38.9)		
Risk factors ^b^				
History of stroke	46 (67.6)	22 (32.4)	0.348	0.555
Hypertension	52 (71.2)	21 (28.8)	3.505	0.061
Diabetes	23 (65.7)	12 (34.3)	0	0.997
Cancer	4 (66.7)	2 (33.3)	0.030	0.958
Paralysis	15 (65.2)	8 (34.8)	0.003	0.957
Atrial fibrillation	9 (64.3)	5 (35.7)	0.014	0.905
Coronary heart disease	9 (81.8)	2 (18.2)	1.424	0.233
Chronic heart failure	12 (60)	8 (40)	0.357	0.550
Pulmonary infection	42 (71.2)	17 (28.8)	1.878	0.171
Laboratory date				
Leukocyte (×10^9^/L) ^c^	8.96 ± 3.18	8.15 ± 3.21	1.220	0.228
Neutrophil (×10^9^/L) ^a^	6.0 (4.30, 9.10)	4.7 (3.40, 8.30)	2.268	0.026 *
Lymphocyte (×10^9^/L) ^a^	1.40 (1.20, 2.00)	1.40 (1.00, 1.90)	1.612	0.113
Monocyte (×10^9^/L) ^c^	0.52 ± 0.23	0.54 ± 0.20	0.444	0.658
Platelets (×10^9^/L) ^c^	226.60 ± 92.27	214.00 ± 76.32	0.690	0.492
CRP (mg/L) ^a^	6.00 (4.80, 18.10)	5.70 (5.20 ± 11.3)	1.458	0.148
Cholesterol (mmol/L) ^c^	4.03 ± 1.05	4.13 ± 0.99	0.442	0.660
Triglyceride (mmol/L) ^a^	1.23 (0.78, 1.53)	1.51 (1.03, 2.40)	2.556	0.014 *
HDL (mmol/L) ^a^	1.04 (0.92, 1.33)	1.10 (0.85, 1.29)	0.016	0.988
LDL (mmol/L) ^c^	2.50 ± 1.16	2.55 ± 1.16	0.211	0.833
HbA1c (≥6.5%) ^b^	19 (61.3)	12 (38.7)	0.510	0.475

GCS—Glasgow Coma Scale; Pre-stroke mRS—pre-stroke modified Rankin Scale; Baseline NIHSS—National Institutes of Health Stroke Scale at admission; TOAST—Trial of Org 10172 in Acute Stroke Treatment; LAA—large-artery atherosclerosis; CE—cardioembolism; CRP—C-reactive protein; HDL—high-density lipoprotein; LDL—low-density lipoprotein; HbA1c—glycosylated hemoglobin. ^a^ represents the Z value analyzed by Mann-Whitney U test, ^b^ is the X^2^ value analyzed by Chi-square test, and ^c^ is the t value analyzed by Student’s *t*-test. * represents *p* < 0.05.

**Table 3:** Univariable and multivariable logistic regression of infarct core prediction in AIS.

Variables	Univariable Models		Full Multivariable Model	
	OR (95% CI)	*p*-Value	OR (95% CI)	*p*-Value
Hypertension	2.311 (0.952~5.613)	0.064	7.179 (1.766~29.186)	0.006 **
Baseline NIHSS (≥15)	2.009 (0.791~5.099)	0.142	1.872 (0.500~7.016)	0.352
Pulmonary infection	1.779 (0.778~4.069)	0.172	1.087 (0.318~3.721)	0.894
Triglyceride	0.447 (0.246~0.813)	0.008 **	0.255 (0.109~0.594)	0.002 **
Iodine density	0.028 (0.007~0.106)	<0.001 **	0.022 (0.003~0.170)	<0.001 **
Zeff	0.093 (0.032~0.271)	<0.001 **	0.869 (0.122~6.177)	0.889

Baseline NIHSS—National Institutes of Health Stroke Scale at admission. Zeff—the effective atomic number. ** represents *p* < 0.01.

## Data Availability

The datasets generated during and/or analyzed during the current study are available from the corresponding author upon reasonable request.

## Supplementary Materials

The following supporting information can be downloaded at: https://www.mdpi.com/article/10.3390/diagnostics13223434/s1, Table S1: Comparison of CTA and CTP imaging scanning parameters in the examination of AIS using dual-layer spectral detector CT.

## Abbreviations

AIS: acute ischemic stroke; IVT: intravenous thrombolysis; EVT: endovascular thrombectomy; CT: computed tomography; CTA: computed tomography angiography; DLCTA: dual-layer spectral detector computed tomography angiography; CTP: CT perfusion imaging; DWI: diffusion-weighted imaging; PET: positron emission tomography; Zeff: effective atomic number; CBV: cerebral blood volume; CBF: cerebral blood flow; MTT: mean transit time; Tmax: time to top; M1-MCA: the M1 segment of the middle cerebral artery; A2-ACA: the A2 segment of the anterior cerebral artery; GCS: Glasgow Coma Scale; mRS: modified Rankin Scale; Baseline NIHSS: National Institutes of Health Stroke Scale at admission; TOAST: Trial of Org 10172 in Acute Stroke Treatment; LAA: large-artery atherosclerosis; CE: cardioembolism; CRP: C-reactive protein; HDL: high-density lipoprotein; LDL: low-density lipoprotein; HbA1c: glycosylated hemoglobin; ROC: receiver operating characteristic; DCA: decision curve analysis.
