# Supplementary material for: Nomogram Based on Dual-Layer Spectral Detector CTA Parameter for the Prediction of Infarct Core in Patients with Acute Ischemic Stroke
Source: Diagnostics (Basel). 2023 Nov 13;13(22):3434. doi: 10.3390/diagnostics13223434 (PMC10670594; doi:10.3390/diagnostics13223434)
Supplement: Supplementary file 1 [file diagnostics-13-03434-s001.zip › diagnostics-2635758-supplementary.pdf]

Table S1. Comparison of CTA and CTP imaging scanning parameters in the examination of AIS using dual-layer spectral detector CT

Parameters	CTA	CTP
Layer thickness (mm)	5	5
Interlayer spacing (mm)	5	5
Collimator width (mm)	40	40
Tube voltage (km)	120	120
Tube current (mAs)	109	40
CTDIvol (mGy)	28.1	103.5
DLP (mGy*cm)	516.3	875

CTDI, CT Dose Index; DLP, dose length product.
